# PD-1抑制剂Nivolumab治疗相关的皮肤毒性1例

**DOI:** 10.3779/j.issn.1009-3419.2019.04.09

**Published:** 2019-04-20

**Authors:** 琳 高, 永峰 虞, 舜 陆

**Affiliations:** 200030 上海，上海交通大学附属胸科医院，上海市肺部肿瘤临床医学中心 Shanghai Lung Cancer Clinical Medical Centers, the Afliated Chest Hospital of Shanghai Jiao Tong University, Shanghai 200030, China

**Keywords:** 免疫相关性皮肤毒性, 肺肿瘤, PD-1抑制剂, Nivolumab, 治疗及预后, Immune-related skin toxicity, Lung neoplasms, PD-1 inhibitor, Nivolumab, Treatment and

## Abstract

**背景与目的:**

Nivolumab是一种与T细胞上的程序性死亡蛋白-1（programmed death-1, PD-1）结合的免疫检查点抑制剂，能够阻断PD-1与程序性死亡蛋白配体（programmed death ligands，包括PD-L1和PD-L2）间的相互作用，从而阻断PD-1通路介导的免疫抑制反应。本研究拟探讨PD-1抑制剂Nivolumab治疗相关的皮肤毒性的临床表现、诊断、治疗和预后。

**方法:**

回顾性分析上海市胸科医院收治的1例晚期肺腺癌患者应用PD-1抑制剂Nivolumab治疗相关的皮肤毒性的临床资料，探讨诊断、治疗和预后分析。结果患者男性，60岁，右肺腺癌术后，术后辅助化疗复发，给予多疗程化疗、靶向治疗、局部骨转移灶放疗后病情仍进展，给予Nivolumab（3 mg/kg/*q2w*，静滴）治疗1个周期后，患者开始出现皮疹并逐渐加重。静脉给予足量类固醇治疗后皮疹好转，类固醇缓慢减量，皮疹控制良好，目前口服类固醇维持治疗，皮疹明显消退。肺内病灶维持稳定。

**结论:**

需要提高对PD-1/PD-L1抑制剂所致免疫相关性皮疹的认识，及早发现，及时处理，预后相对良好。

## Abstract (en)

**Background and objective:**

Nivolumab is an checkpoint inhibitor combining with programmed death-1 (PD-1) receptor on T cells, which can block the interactions between PD-1 and programmed death ligands (PD-L), including PD-L1 and PD-L2. And then block the immunosuppression mediated by the PD-1 pathway. The aim of the study is to investigate the clinical manifestations, diagnosis, treatment and prognosis of treatment-related skin toxicity caused by PD-1 inhibitor Nivolumab.

**Methods:**

The clinical data of treatment-related skin toxicity caused by PD-1 inhibitor Nivolumab in a patient with advanced lung adenocarcinoma admitted to the Shanghai Chest Hospital was retrospectively analyzed. The diagnosis, treatment and prognosis of the patient were discussed. Results The patient was a 60-year-old male presented with relapse after surgery and adjuvant postoperative chemotherapy for his lung carcinoma. The patient's condition still progressed after multiple chemotherapy, targeted therapy and local radiotherapy of bone metastasis. Then Nivolumab, a kind of PD-1 inhibitors, was given intravenously every 3 weeks with the average dosage 3 mg/kg. After one cycle of Nivolumab, the patient began to have skin rashes, which aggravated gradually. The patient's skin toxicity was alleviated after enough steroids and was controlled with tapering steroids slowly. Now the patient was still given oral steroids treatment. And the lung disease remained stable.

**Conclusion:**

Immunerelated skin toxicity associated with PD-1 inhibitor should be aware of; early detection, early treatment and the prognosis could be better. It is necessary to improve the understanding of Immune-related skin toxicity associated with PD-1 inhibitor, to diagnose and treat it early, and the prognosis could be better.

程序性死亡蛋白-1（programmed death-1, PD-1）是免疫系统效应器阶段的检查点，属于CD-28/CTLA-1家族的一个免疫受体。它能够通过与程序性死亡蛋白配体（programmed death ligand, PD-L），即PD-L1或PD-L2这两种配体相互作用，募集蛋白酪氨酸磷酸酶SHP-2，负性调控PD-1信号通路。由于体内PD-L分布广泛，该信号通路受阻影响到免疫反应各个方面，包括自身免疫、肿瘤免疫、感染免疫、移植免疫、过敏等^[1]^。继化疗、靶向治疗之后，免疫治疗成为治疗非小细胞肺癌的新手段，并且疗效显著，其中包括靶向程序性死亡蛋白-1及其配体（PD-1/PD-L1）的单克隆抗体。Nivolumab是一种PD-1抑制剂，能够通过直接阻断PD-1而增强已经存在的免疫应答，包括抗肿瘤应答^[2]^。尽管患者对免疫治疗的耐受性很好，但它们有一种不同于传统化疗的副反应，即免疫相关副反应，早期识别和治疗非常关键^[3]^。Nivolumab能够引起多种免疫相关副反应，比如皮疹、肝炎、肺炎、结肠炎、心肌炎等。据报道，接受Nivolumab治疗的非小细胞肺癌患者，皮肤毒性发生率接近10%甚至更高^[4]^。由于PD-1抑制剂的应用在国内刚开始，因此PD-1抑制剂治疗国内人群的疗效及副反应等方面知之甚少。在此，我们报道了1例接受Nivolumab免疫治疗的肺腺癌患者发生严重免疫相关性皮肤毒性，提醒我们需要提高对PD-1/PD-L1抑制剂所致免疫相关皮疹的认识，及早发现，及时处理，预后相对良好。

## 1 临床资料

患者男性，60岁，体质量70 kg。2010年6月体检发现血癌胚抗原（carcinoembryonic antigen, CEA）升高至9.5 ng/mL，胸部计算机断层扫描（computed tomography, CT）提示右肺结节。正电子发射计算机断层显像（positron emission tomography-CT, PET-CT）提示右下肺内基底段分叶状结节，大小约2.2 cm×2.6 cm。2010年11月行全麻下右肺下叶切除术+淋巴结清扫术，病理：右肺下叶后基底段肿块2.5 cm×2.5 cm×2 cm，腺癌，乳头状亚型，中分化，侵脏层胸膜，支气管切端未见癌转移。肺门组淋巴结（1^+^/2）见癌转移，气管旁组淋巴结（0/1）、气管支气管组淋巴结（0/2）、隆突下组淋巴结（0/2）、下肺韧带组淋巴结（0/1）、叶间组淋巴结（0/3）、上中下叶组淋巴结（0/1）、上叶管口淋巴结（0/2）未见癌转移。术后分期：p-T2N1M0 IIb期。肿瘤组织基因检测：EGFR Exon 19 del L747-T75，*ALK*-。2010年12月行培美曲塞+顺铂方案辅助化疗。考虑患者出现3度胃肠道反应，2011年1月起改为培美曲塞+卡铂化疗3个周期。定期复查随访未见疾病进展。2015年12月胸部CT提示右侧胸膜及叶间胸膜新发多处结节影，考虑胸膜转移；右侧胸腔新发胸水。2016年2月开始口服易瑞沙。2016年2月胸部CT提示右侧胸膜及叶间膜多处小结节影较前缩小。2017年5月复查血CEA升高至150 ng/mL，胸部CT提示残肺及胸膜多发小结节，考虑转移瘤；左肺下叶小结节，右肺少量胸腔积液。提示疾病进展，遂停止口服易瑞沙。2017年5月起行培美曲塞+顺铂方案化疗4次，血CEA降至23.1 ng/mL。2017年8月、9月、10月随访病情稳定。2017年12月血CEA 27.9 ng/mL，结合胸部CT，考虑病情进展。2017年12月行培美曲塞+卡铂化疗2次。2018年4月骨扫描提示左侧颌面部、左侧坐骨放射性浓聚灶，右侧第一肋放射性摄取稍高。2018年5月复查血CEA 78.9 ng/mL，CT提示右侧胸膜转移灶较前增大、增多，MRI提示左侧坐骨异常信号，考虑转移可能大。考虑疾病进展。2018年6月行左侧坐骨转移灶SBRT治疗，DT：30 Gy/3 Fx。2018年6月CT引导下右侧胸膜转移灶穿刺活检术，病理提示腺癌。2018年8月29日患者行第1周期Nivolumab 200 mg免疫治疗，治疗后1度皮疹，嘱患者避免日晒，给予尿素乳膏外用。2018年9月13日行第2周期Nivolumab 200 mg免疫治疗，治疗后仍有1级手足皮疹，伴瘙痒，给予氯雷他定口服，继续尿素乳膏外用。2018年9月27日患者行第3周期Nivolumab 200 mg免疫治疗，治疗后患者仍为1度手足皮疹，伴瘙痒。2018年10月11日患者行第4周期Nivolumab 200 mg免疫治疗，治疗后出现双手及下肢皮疹（图 1A），逐渐加重至3度（图 1B），伴瘙痒疼痛，伴水泡，易破裂出血形成溃疡，伴口腔溃疡。患者查体未见淋巴结肿大，无发热，EBV、CMV检查阴性排除病毒感染，免疫球蛋白水平和补体水平正常排除免疫缺陷、系统性红斑狼疮、结缔组织病等自身免疫性疾病，结合患者近期用药情况，诊断考虑Nivolumab所致免疫相关性皮疹可能性大。2018年10月29日起给予注射用甲泼尼龙琥珀酸钠80 mg/d静脉滴注，每隔1周减量20 mg/d直至40 mg/d静脉滴注，同时继续口服氯雷他定，控制血糖，辅以兰索拉唑抑酸护胃，静脉补充维生素C，结合硼酸冲洗，保持创面清洁干燥，治疗3周后皮疹明显好转，减轻至1度（图 2）。改为强的松30 mg/d口服，同时口服耐信20 mg/d，维持1周后减量为15 mg/d口服，2周后减量为10 mg/d，10 mg/d维持3周后减量至5 mg/d至今。门诊随诊至今，未再应用免疫治疗，皮疹仍稳定为1度（图 1C）。2018年12月3日复查胸部增强CT提示肺内病灶稳定（图 3）。

**1:**
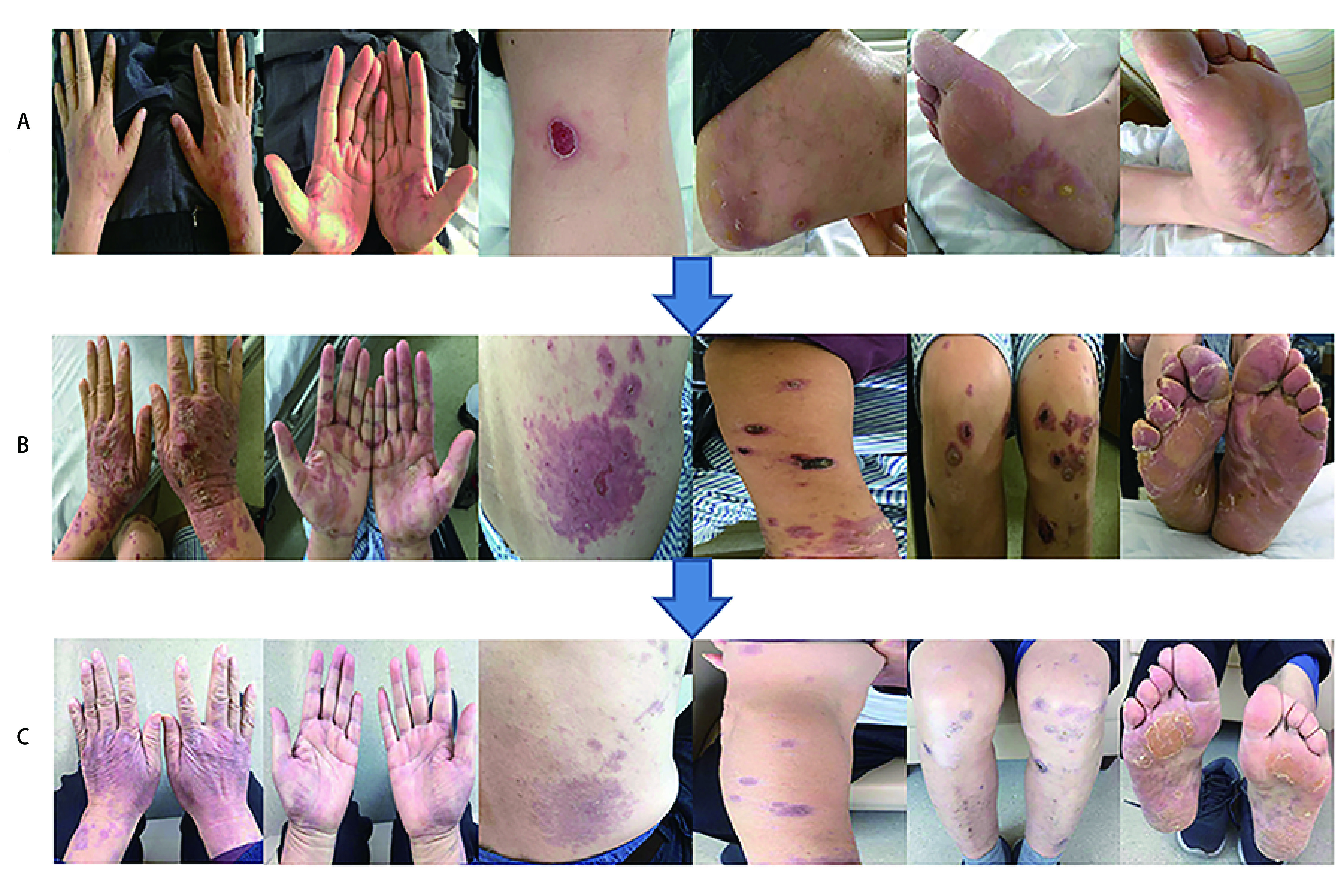
类固醇治疗前后全身皮疹的变化情况。A：4周期Nivolumab免疫治疗后出现1度皮疹，覆盖 < 10%体表面积，伴随瘙痒和红斑；B：注射用甲泼尼龙琥珀酸钠治疗第9天皮疹达顶峰至3度，覆盖了20%体表面积，并伴随瘙痒、红斑和表皮剥脱；C：类固醇治疗第51天，患者皮疹维持在1度皮疹状态。皮疹覆盖体表面积小于 < 10%，伴有红斑，不伴瘙痒和表皮剥脱。 The changing status of body rashes before and after the steroids treatment. A: Grade 1 skin rashes appeared after 4 doses of Nivolumab treatment, it covered < 10% body surface area with pruritus and erythema; B: Skin rashes gradually aggravated to grade 3 on the 9^th^ day during methylprednisolone treatment, it covered 20% body surface area with pruritus, erythema and epidermal detachment; C: The present skin reaction has reverted to grade 1 on the 51^th^ day of steroids treatment. Skin rashes covered < 10% body surface area, with erythema, but without pruritus and epidermal detachment.

**2:**
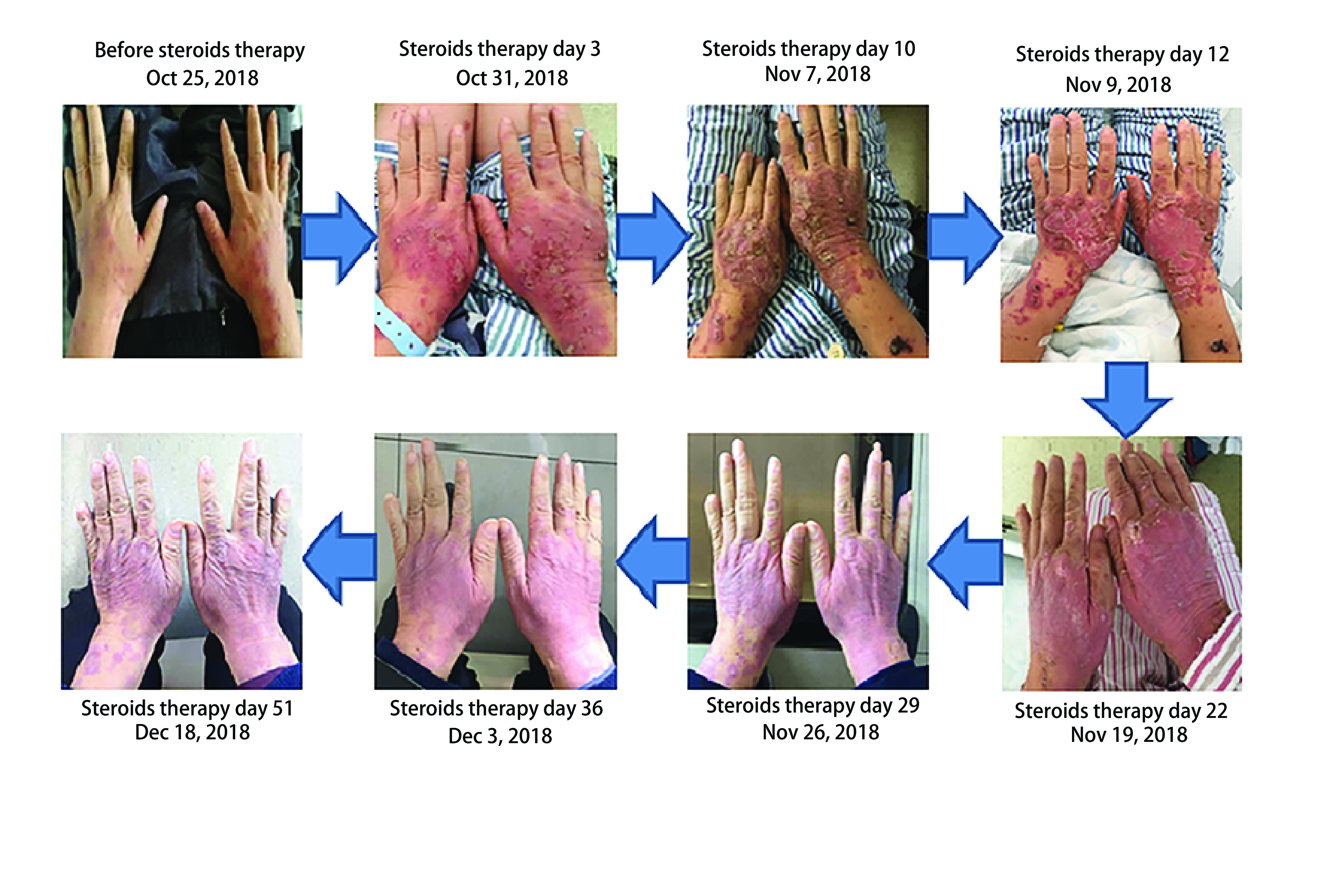
类固醇治疗前后手部皮疹的变化情况。随着时间推移，手部皮疹进行性加重至3度皮疹后逐渐消退并维持1度皮疹，其他部位的皮疹也是如此。 The changing status of hand skin rashes before and after the steroids treatment. Over time, the hand skin rashes aggravated to grade 3, then gradually alleviated to grade 1, as well as the rashes of other parts of the body.

**3:**
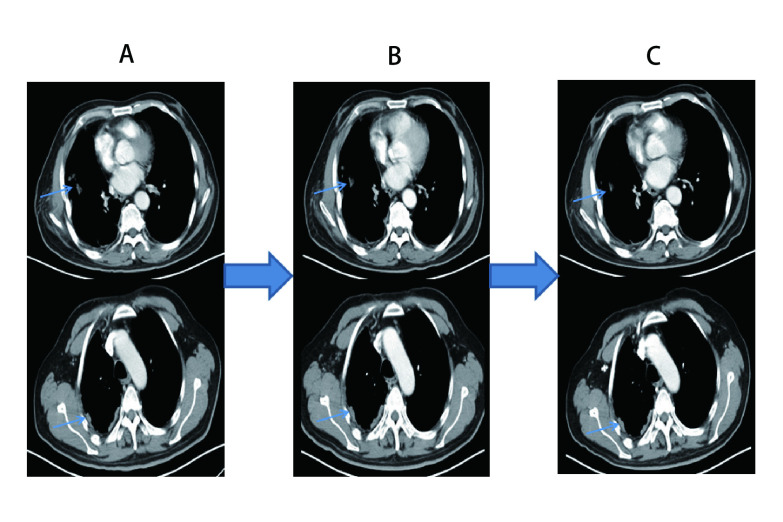
Nivolumab治疗前后的胸部CT表现。A：应用Nivolumab治疗前，胸部CT显示右肺、右侧胸膜多发转移；B：应用3次Nivolumab治疗后胸部CT显示转移灶稳定；C：停用Nivolumab免疫治疗7周，胸部CT显示疾病稳定，右肺、右侧胸膜转移灶较前稳定略缩小。 Chest CT scans before and after Nivolumab. A: The chest CT scans before Nivolumab at different layers showed multiple metastases in right lung and pleura; B: The chest CT scans after 3 doses of Nivolumab treatment revealed the metastases remained stable; C: After discontinuation of Nivolumab treatment for 7 weeks, the chest CT scans showed that the metastatic nodules were stable and slightly smaller than those before.

## 2 讨论

肿瘤细胞能够表达PD-L1，与效应T细胞上的PD-1/B7.1结合，抑制T细胞功能，发生免疫逃逸，而PD-1抑制剂能够阻断该信号通路^[5]^。靶向PD-1的单克隆抗体是治疗多种肿瘤的一种新方法，已经成为美国国立综合癌症网络（National Comprehensive Cancer Network, NCCN）指南中转移性非小细胞肺癌的标准治疗方案^[6]^。

尽管这些PD-1抑制剂耐受性良好，但它们可能引起一种新的副反应，即免疫相关不良反应，可累及多器官系统^[7]^。因此，监测和管理免疫相关副反应至关重要。应用Nivolumab免疫治疗，皮肤毒性最先发生，其次是结肠炎，最后发生的是肝炎和内分泌紊乱^[8]^。而皮疹和瘙痒是最常见的免疫相关皮肤毒性。在Nivolumab单药免疫治疗的副反应中，皮疹发生率最高，接近40%，而Nivolumab联合Ipilimumab免疫治疗中，这一数据接近60%^[9]^。幸运地是，3级-4级皮疹发生率低于10%^[10]^。免疫相关皮肤毒性通常发生在免疫治疗几天或几周内^[11]^，有的也可能延迟发生在免疫治疗几个月之后^[12]^。大部分皮肤毒性级别较低并且能够控制，但是也可能发生危及生命的剥脱性皮肤病，比如Stevens-Johnson综合征/中毒性表皮坏死松解症（Stevens-Johnson syndrome/toxic epidermal necrolysis, SJS/TEN）和药物性皮疹伴嗜酸性粒细胞增多伴全身症状（drug rash with eosinophilia and systemic symptoms, DRESS）^[13]^。

根据欧洲肿瘤内科学会（European Society for Medical Oncology, ESMO）指南，当患者应用免疫检查点抑制剂后出现皮肤毒性，首先应该排除其他皮肤疾病，如感染，其他药物引起的皮肤毒性和系统性疾病引起的皮肤毒性等。其次，需要通过体格检查，一般情况如发热、淋巴结肿大、血细胞计数、肝肾功能等检查评估皮肤毒性严重程度，以上有助于排除SJS/TEN和DRESS等某些可能致死性皮肤毒性。一旦发生上述危及生命的皮肤毒性，患者应永久停用免疫检查点抑制剂，需要住院治疗，并在皮肤科医生指导下或在皮肤科进行对症治疗。对于皮疹的分度及处理如下所示。1度：皮疹覆盖 < 10%的体表面积，伴或不伴有伴随症状（如瘙痒、烧灼感和紧绷感等）。对于1度皮疹，完善相关检查排除其他原因所致皮疹。可继续应用免疫治疗，避免皮肤刺激和日晒，推荐应用局部润肤剂，可局部应用弱效类固醇软膏，如有瘙痒可口服或局部应用抗组胺药物。2度：皮疹覆盖10%-30%的体表面积。对于2度皮疹，继续应用免疫治疗，完善检查同1度皮疹。考虑皮肤科会诊和皮肤活检。支持治疗同1度皮疹。对症处理包括局部应用弱效或强效类固醇软膏每天2次，如有瘙痒口服或局部应用抗组胺药物。3度：皮疹覆盖 > 30%的体表面积，或2度皮疹伴大量的伴随症状。对于3度皮疹，暂停免疫治疗，检查后排除其他原因皮疹。请皮肤科会诊，考虑皮肤活检和临床摄影。局部应用强效类固醇软膏，其他局部治疗同前。开始应用类固醇激素治疗，如轻至中度皮疹，应用0.5 mg/kg-1 mg/kg泼尼松龙3天，每天1次，并在1周-2周内减量。如皮疹严重，静脉应用注射用甲泼尼龙琥珀酸钠0.5 mg/kg-1 mg/kg，改善后改为口服类固醇激素，2周-4周内逐渐减量。皮疹减退至1度和轻度2度时，与患者和医师讨论后可重新开始免疫治疗。4度：皮肤蜕皮 > 30%的体表面积，合并伴随症状（如红斑、紫癜、表皮剥脱）。对于4度皮疹，完善检查排除其他疾病所致皮疹，请皮肤科急会诊，皮肤活检和临床摄影。停止免疫治疗，静脉应用注射用甲泼尼龙琥珀酸钠1 mg/kg-2 mg/kg^[14]^。

本文中的患者在应用4周期Nivolumab免疫治疗后，发生3度免疫相关皮肤毒性，根据指南推荐，暂停免疫治疗药物，排除其他原因所致皮疹，如病毒性疾病、感染、其他药物引起的药疹等，并请皮肤科会诊指导用药。给予注射用甲泼尼龙琥珀酸钠80 mg/d静滴，同时口服氯雷他定，外用硼酸冲洗并暴露创面，抑酸护胃，补充维生素C。在3周内注射用甲泼尼龙琥珀酸钠逐渐减量，皮疹改善后改为口服强的松维持，用药后患者皮疹逐步好转并维持1度。

综上所述，鉴于国内Nivolumab新近上市，国内对该药用药经验比较欠缺。本文提供1例Nivlumab导致免疫性皮疹的治疗和转归经过，早期诊断、及早干预至关重要，可能为国内其他临床医师提供参考。
